# Effects of 3 Weeks of Oral Low-Dose Cobalt on Hemoglobin Mass and Aerobic Performance

**DOI:** 10.3389/fphys.2018.01289

**Published:** 2018-09-19

**Authors:** Torben Hoffmeister, Dirk Schwenke, Oliver Krug, Nadine Wachsmuth, Hans Geyer, Mario Thevis, William C. Byrnes, Walter F. J. Schmidt

**Affiliations:** ^1^Department of Sports Medicine and Sports Physiology, University of Bayreuth, Bayreuth, Germany; ^2^Institute of Doping Analysis und Sports Biochemistry, University of Dresden, Dresden, Germany; ^3^Institute of Biochemistry, German Sport University, Cologne, Germany; ^4^Department of Integrative Physiology, University of Colorado, Boulder, CO, United States

**Keywords:** erythropoietin, aerobic performance, doping, blood manipulation, supplement, sports nutrition

## Abstract

**Introduction:** Cobalt ions (Co^2+^) stabilize HIFα and increase endogenous erythropoietin (EPO) production creating the possibility that Co^2+^ supplements (CoSupp) may be used as performance enhancing substances. The aim of this study was to determine the effects of a small oral dosage of CoSupp on hemoglobin mass (Hbmass) and performance with the objective of providing the basis for establishing upper threshold limits of urine [Co^2+^] to detect CoSupp misuse in sport.

**Methods:** Twenty-four male subjects participated in a double-blind placebo-controlled study. Sixteen received an oral dose of 5 mg of ionized Co^2+^ per day for 3 weeks, and eight served as controls. Blood and urine samples were taken before the study, during the study and up to 3 weeks after CoSupp. Hbmass was determined by the CO-rebreathing method at regular time intervals, and VO_2max_ was determined before and after the CoSupp administration period.

**Results:** In the Co^2+^ group, Hbmass increased by 2.0 ± 2.1% (*p* < 0.001) while all the other analyzed hematological parameters did not show significant interactions of time and treatment. Hemoglobin concentration ([Hb]) and hematocrit (Hct) tended to increase (*p* = 0.16, *p* = 0.1) and also [EPO] showed a similar trend (baseline: 9.5 ± 3.0, after 2 weeks: 12.4 ± 5.2 mU/ml). While mean VO_2max_ did not change, there was a trend for a positive relationship between changes in Hbmass and changes in VO_2max_ immediately after CoSupp (*r* = 0.40, *p* = 0.11). Urine [Co^2+^] increased from 0.4 ± 0.3 to 471.4 ± 384.1 ng/ml (*p* < 0.01) and remained significantly elevated until 2 weeks after cessation.

**Conclusion:** An oral Co^2+^ dosage of 5 mg/day for 3 weeks effectively increases Hbmass with a tendency to increase hemoglobin concentration ([Hb]) and hematocrit (Hct). Because urine Co^2+^ concentration remains increased for 2 weeks after cessation, upper limit threshold values for monitoring CoSupp can be established.

## Introduction

From the mid-1940s until the 1980s, cobalt ions (Co^2+^) were used for patients experiencing anemia from various causes (Jelkmann, 2012; Ebert and Jelkmann, 2014). Co^2+^ acts similarly as a hypoxic stimulus; it stabilizes HIF-1α and HIF-2α and thereby increases plasma erythropoietin concentration ([EPO] (for a review, see Jelkmann, 2012). Because of the severe side effects of high-dose Co^2+^ treatment, it was replaced by anabolic steroids and later by recombinant erythropoietin (Ebert and Jelkmann, 2014).

Maximum oxygen uptake as a measure of endurance performance is closely related to the absolute amount of circulating hemoglobin. After phlebotomy or after application of rhEPO, a change of 1 g of hemoglobin causes a change in VO_2max_ of approx. 4 ml/min (Schmidt and Prommer, 2010). This is why blood manipulation, either by erythropoietic agents or by blood transfusions, occurs widely as a malpractice within elite endurance sports (Sottas et al., 2011; Wintermantel et al., 2016). CoSupp could also be used to stimulate endogenous EPO production and to increase endurance performance (Lippi et al., 2005, 2006). In recent years, CoSupp has been marketed as a nutritional supplement for endurance athletes with unsubstantiated performance enhancing effects (Thevis et al., 2016). It is therefore no surprise that CoSupp has been discussed among athletes, and in 2015, World Anti-Doping Agency (WADA) included CoSupp in its prohibited list (WADA, 2017). Until now, however, no threshold values for urine or blood indicating CoSupp use have been established.

Because small amounts of Co^2+^(~0.1 μg/day) are an essential co-factor for vitamin B12 (Tvermoes et al., 2013, 2014), Co^2+^ is a necessary component of our daily nutrition. Co^2+^ is detectable in blood (Tvermoes et al., 2014) and urine (Krug et al., 2014), but the concentrations are very low and close to the detection limits. To create upper threshold limits in blood and urine for the detection of CoSupp misuse, the amount necessary for erythropoietic stimulation must be known. For this purpose, earlier studies are not helpful, as they used very high dosages (mostly > 25 mg Co^2+^/day) (Bradberry et al., 1989; Finley et al., 2012), which increase the risk of acute poisoning and organ injury, such as gastrointestinal sickness, thyroid dysfunction, myocardial effects and neural disturbances (Ebert and Jelkmann, 2014). Studies using low-dose oral CoCl_2_ in healthy volunteers at 0.45 mg Co^2+^/day for 2 weeks (Tvermoes et al., 2013) and 1 mg Co^2+^/day for 90 days (Tvermoes et al., 2014) did not yield hematological changes, with the exception of a decrease in ferritin during the 90-day period.

In a recent study, the minimum amount of daily oral Co^2+^ administration leading to increased plasma [EPO] was determined (Hoffmeister et al., 2018). A single dosage of 10 mg in moderately trained young men resulted in a 53% increase in [EPO], and a dosage of 10 mg for 5 days resulted in a 50% increase in immature reticulocyte fraction and 12% decrease in ferritin concentration. A dosage of 5 mg Co^2+^ increased plasma [EPO] by 20% which can be assumed to be closer to the erythropoietic threshold than 10 mg per day. Long-term administration of this dosage may be effective for increasing hemoglobin mass and improving endurance performance.

The aim of this study was to administer a 3-week daily 5 mg Co^2+^ treatment to healthy leisure athletes and investigate the effects on hemoglobin mass and aerobic performance.

## Methods

### Subjects

In total, 24 healthy male non-smoking subjects participated in the study. Eight received a placebo and 16 received the Co^2+^ supplement. The subjects were recruited by a notice shown to sports students at the University of Bayreuth. The project was approved by the ethics committee of the University of Bayreuth, Germany. All subjects were informed in verbal and written form about the aim of the study, the possible risks, and the possibility to withdraw without any personal consequences, and they gave their written informed consent. The anthropometric data of the test subjects are presented in Table 1.

**Table 1:** Anthropometric characteristics of the test subjects.

	***n* =**	**Age (years)**	**Height (cm)**	**Body mass (kg)**	**BMI**
Placebo	8	22.4 ± 2.0	181.1 ± 5.1	77.7 ± 5.1	23.7 ± 1.4
Cobalt	16	22.9 ± 2.6	179.9 ± 6.2	75.0 ± 8.8	23.1 ± 2.0

### Design of the study

The study was performed using a double blind, placebo-controlled design consisting of a 1-week baseline period, a 3-week supplementation period and a 3-week post-treatment period. Sixteen subjects received a daily oral dosage of CoSupp containing 5 mg Co^2+^, and eight of the subjects received a placebo.

Venous blood and urine samples were taken before (duplicate measures) during (once per week) and after (immediately and once per week for 3 weeks) the treatment. Each blood sample included 3 ml for whole blood parameters and 9 ml for serum parameters while urine samples consisted of 50 ml collected in the morning.

Hemoglobin mass (Hbmass) was determined before (duplicate measures), during and after the treatment period. An aerobic performance test (incremental step test) on a cycle ergometer was performed before, the day after and 1 week after the treatment period.

### Cobalt supplementation

A commercially available dietary CoSupp (Minerallife, Colorado Springs, CO, USA) was used after its Co^2+^ content was checked in the WADA-accredited Institute of Biochemistry of the German Sport University, Cologne, Germany. Besides ionized cobalt (Co^2+^) listed additional ingredients were carbohydrate derived fulvic acid, natamycin, and nisin which were included for their anti-bacterial and anti-fungal properties.

The real Co^2+^ concentration of the liquid supplement markedly deviated from the data provided by the manufacturer (0.2 mg/ml) and ranged between 0.24 and 0.86 mg/ml in different batches. The amount of Co^2+^ administered to our subjects was, therefore, calculated from our own results and not from the manufacturer's information. The subjects received their CoSupp in a mixture of orange juice and buttermilk partitioned in five doses every 4 h (except in the morning at 4 am) for 3 weeks. The eight control subjects received a placebo, orange juice with buttermilk, at the identical times. All subjects were regularly questioned as to whether they were experiencing any side effect. No subject reported any side effect during and for 3 weeks after the treatment.

### Sample transport and storage

The whole procedure, including urine collection, blood sampling, sample transport, sample storage and sample analyses, was performed according to current WADA guidelines (WADA, 2016). The blood samples were taken in the morning between 8 and 9 a.m. after leaving the subject for at least 15 min in a sitting position. Within 24 h, the samples were transported under monitored cool conditions to the WADA-accredited Institute of Doping Analysis and Sports Biochemistry in Dresden, Germany. Urine samples were taken 15–20 min before blood sampling, and an aliquot of 50 ml was also transported under the same standardized conditions to the Institute of Biochemistry of the German Sport University, Cologne, Germany, where samples were stored at −80°C until they were analyzed.

### Urine and blood analytical procedures

For cobalt ion analysis, urine samples were subjected to an EN ISO 17294-accredited test method for the quantitative determination of cobalt in an aqueous and urinary matrix using inductively coupled plasma-mass spectrometry (ICP-MS). In brief, urine aliquots were diluted with aqueous HNO_3_ and subjected to ICP-MS analysis using a Thermo (Bremen, Germany) Element 2 magnetic sector or XSERIES 2 apparatus. The method's limit of quantification (LOQ) was 0.5 ng/mL (see Krug et al., 2014).

The Sysmex XT2000i hematological analyzer (Sysmex, Norderstedt, Germany) was used for the determination of hemoglobin concentration ([Hb]), hematocrit (Hct), reticulocyte number, immature reticulocyte fraction (IRF), red cell indices and other routine hematological parameters.

In serum, the following parameters were determined: ferritin [LKFE1, ELISA & Immulite 1000 (Siemens Healthcare Diagnostics GmbH, Germany)], erythropoietin (EPO) [LKEP1, ELISA & Immulite 1000 (Siemens Healthcare Diagnostics GmbH, Germany)], soluble transferrin receptor (sTFR) [Quantikine IVD (DTFTR1) Assay (R&D Systems, bio-techne, Wiesbaden, Germany) & Infinite 200 PRO (Tecan, Switzerland)], and C-reactive protein (CRP) [high sensitive–LKCRP1, ELISA & Immulite 1,000 (Siemens Healthcare Diagnostics GmbH, Germany)].

### Hemoglobin mass determination

Hemoglobin mass was determined by using the optimized CO-rebreathing method as described and modified by Schmidt and Prommer (Schmidt and Prommer, 2005; Prommer and Schmidt, 2007). Briefly, a bolus of 99.97% carbon monoxide (CO; 1.0 ml CO per kg body mass) was administered to subjects and rebreathed along with 3 liters of 100% O_2_ for 2 min. Arterialized capillary blood samples were taken from a hyperemized earlobe before and 7 min after the rebreathing procedure and analyzed in sextuplicate using an OSM3 hemoximeter (Radiometer, Denmark). End tidal [CO] was assessed before and 2 min after the rebreathing procedure using a portable CO detector (Draeger Pac7000, Germany). Hbmass was assessed in duplicate prior to the intervention; there was no significant difference between the 2 baseline tests (difference: 7.4 ± 19.5 g, *n* = 24) so the mean of each subject's 2 tests was used as their baseline value. Typical error for Hbmass determined from these duplicate measurements was 1.6%.

### Aerobic performance determination

Aerobic performance was determined on a cycle ergometer (Lode, Groningen, Netherlands) by starting with 100 watts (3 min) and increasing the load by 50 watts every 3 min (this occurred stepwise every minute by steps of 17, 17, and 16 Watts) until subjective exhaustion. Key dependent variables were VO_2max_ (METALYZER® 3B, Cortex, Leipzig Germany), time until exhaustion, power at exhaustion, and sub-maximal power at blood lactic acid concentrations of 2 mmol/l and 4 mmol/l. Blood samples for lactic acid determination were taken from a hyperemized earlobe before exercise, every 3 min during exercise plus immediately and 1, 3, 5, and 7 min after exhaustion. The typical error for determination of VO_2max_ in our laboratory is 2.5%.

### Statistics

Data are presented as the mean ± standard deviation (SD). An analysis of variance with repeated measurements was carried out to detect main effects for group and time as well as group by time interactions. Significance level was set at *p* < 0.05. In case of significant outcome of the ANOVA (interaction of time and treatment), unpaired *t*-tests were used to compare mean values for both groups at identical time points, and paired *t*-tests were used to compare the mean values of identical individuals at different time points. Linear regression analyses were performed to detect any relationship between VO_2max_ and Hbmass.

## Results

### Cobalt excretion in urine

**Figure 1:**
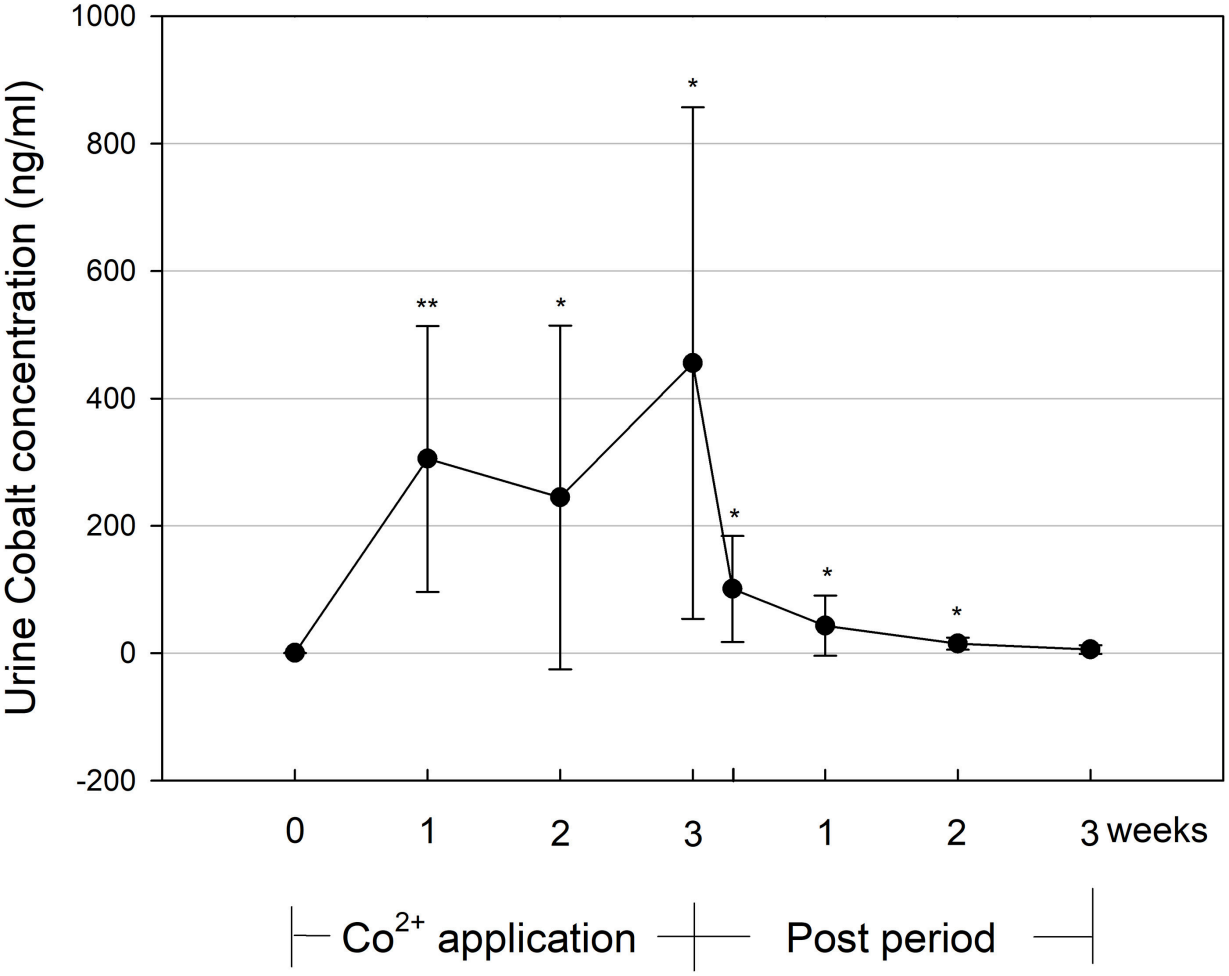
Urine cobalt concentration during and until 3 weeks after the 3-week Co^2+^ administration period. Significant changes from baseline values: ^*^p < 0.05, ^**^p < 0.01.

Urine cobalt concentration (Figure 1) was elevated with CoSupp during the 3-week Co^2+^ application period. Mean values of 305 ± 209 ng/ml (significance against baseline: *p* < 0.01) were found after week 1 and significantly increased to 456 ± 402 ng/ml (*p* < 0.01) by the end of week 3. During the post-treatment period values decreased over time but did not return to baseline until 2 weeks after the treatment. It should be noted that there was a large intra- (mean STD = 172 ng/ml) and inter- (mean STD = 254 ng/ml) subject variability in urine cobalt concentration.

### Changes in hematological parameters

**Figure 2:**
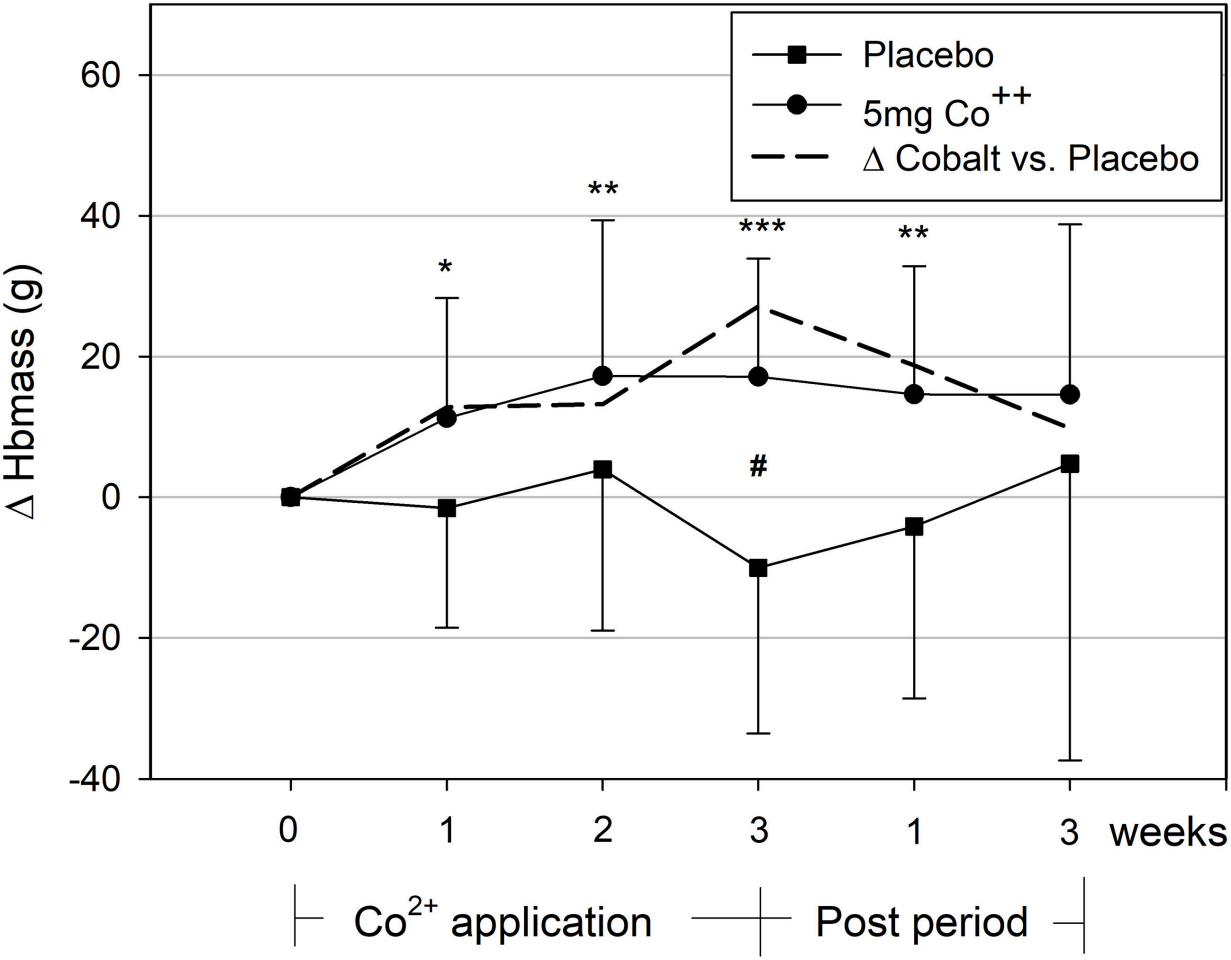
Changes in hemoglobin mass during and after the 3-week cobalt application period. Significant changes from baseline values: ^*^, ^**^, ^***^p < 0.05, 0.01, 0.001; between groups: #*p* < 0.05. The dashed line indicates the difference between cobalt and placebo groups.

The comparison of Hbmass of the cobalt and placebo groups yielded a significant treatment by time interaction (*p* < 0.05). For the cobalt group, *post-hoc* tests showed significant increases in Hbmass from the first week of treatment until 1 week thereafter (Figure 2).

Ferritin showed a clear time effect with decreasing concentrations in both groups without an interaction of time and treatment (Table 2).

**Table 2:** Changes in serum EPO, TfR, ferritin, and CRP concentrations between the cobalt vs. placebo groups before, during, and after cobalt administration.

			**Cobalt administration**				**Post-administration period**		
		**Baseline**	**7 days**	**14 days**	**21 days**	**2 days**	**7 days**	**14 days**	**21 days**
EPO (mIU/ml)	Cobalt	9.5 ± 3.0	10.1 ± 2.9	12.4 ± 5.2	10.6 ± 3.5	10.8 ± 4.4	10.9 ± 6.2	12.6 ± 4.5	11.6 ± 4.1
	Placebo	9.8 ± 1.7	9.6 ± 2.0	10.2 ± 2.7	11.3 ± 2.8	10.2 ± 3.2	10.3 ± 3.0	10.2 ± 3.3	14.5 ± 4.2
sTfR (nmol/l)	Cobalt	27.8 ± 5.2	28.1 ± 6.6	31.7 ± 6.0	29.8 ± 4.8	30.9 ± 6.9	30.5 ± 6.3	30.9 ± 5.5	29.5 ± 9.5
	Placebo	33.4 ± 4.6	32.7 ± 3.6	33.6 ± 6.2	31.6 ± 5.2	33.5 ± 7.4	35.8 ± 8.5	29.9 ± 8.5	32.8 ± 5.6
Ferritin (ng/ml)	Cobalt	108 ± 46	94 ± 42^*^	92 ± 40^*^	89 ± 46^**^	88 ± 42^**^	89 ± 44^*^	94 ± 37^*^	98 ± 47
	Placebo	108 ± 49	93 ± 53^*^	95 ± 51	100 ± 43	92 ± 38	92 ± 38	108 ± 42	97 ± 57
CRP (mg/l)	Cobalt	2.6 ± 4.9	1.4 ± 1.8	2.0 ± 2.3	1.5 ± 2.4	1.2 ± 1.7	2.1 ± 4.8	3.4 ± 6.4	2.6 ± 3.5
	Placebo	0.6 ± 0.3	1.1 ± 1.1	0.8 ± 0.5	0.6 ± 0.7	0.5 ± 0.4	0.4 ± 0.3	0.8 ± 1.1	0.6 ± 0.3

Presented are means ± standard deviations (SD). EPO, erythropoietin; sTfR, serum transferrin receptor, and CRP, C-reactive protein. Significance of differences from baseline (post-hoc tests):

*, ** *p < 0.05, 0.01*.

All of the other parameters were not significantly affected by CoSupp as indicated by non-significant interaction of time and treatment. There were, however, trends in the cobalt group for increased [Hb] (*p* = 0.16) and Hct (*p* = 0.10; Table 3). Also [EPO] tended to be higher after 2 weeks of CoSupp.

**Table 3:** Changes in hematological parameters between the cobalt vs. placebo groups before, during, and after cobalt administration.

			**Cobalt administration**			**Post-administration period**			
		**Baseline**	**7 days**	**14 days**	**21 days**	**2 days**	**7 days**	**14 days**	**21 days**
Hbmass (g)	Cobalt	839 ± 106	851 ± 120^*^	857 ± 113^**^	856 ± 110^***^.^#^		854 ± 112^**^		838 ± 67
	Placebo	884 ± 116	883 ± 123	888 ± 132	874 ± 124		880 ± 119		916 ± 136
[Hb] (g/100 ml)	Cobalt	15.1 ± 0.9	15.3 ± 1.0	15.0 ± 1.1	15.2 ± 1.3	15.0 ± 1.0	15.1 ± 1.0	14.7 ± 0.9	15.1 ± 0.9
	Placebo	15.1 ± 0.9	14.9 ± 1.0	15.2 ± 0.8	14.9 ± 1.0	14.8 ± 0.8	15.0 ± 1.2	14.8 ± 1.0	14.9 ± 1.0
Hct (%)	Cobalt	43.3 ± 2.3	44.3 ± 2.5	43.3 ± 3.0	43.8 ± 3.5	43.5 ± 2.6	43.8 ± 2.8	42.8 ± 2.6	43.5 ± 2.8
	Placebo	44.1 ± 2.5	43.7 ± 2.5	44.1 ± 2.2	43.7 ± 2.7	43.0 ± 2.6	43.9 ± 3.5	43.0 ± 3.1	43.6 ± 3.10
RBC (10^6^/μl)	Cobalt	5.00 ± 0.30	5.07 ± 0.29	4.96 ± 0.30	5.03 ± 0.43	4.97 ± 0.39	5.01 ± 0.29	4.88 ± 0.37	4.97 ± 0.39
	Placebo	4.95 ± 0.31	4.89 ± 0.34	4.97 ± 0.29	4.90 ± 0.38	4.84 ± 0.38	4.93 ± 0.39	4.85 ± 0.41	4.93 ± 0.31
MCV (fl)	Cobalt	87.0 ± 4.6	87.6 ± 4.3	87.5 ± 4.4	87.4 ± 4.5	87.6 ± 4.2	87.6 ± 3.5	88.1 ± 3.2	87.7 ± 3.7
	Placebo	89.2 ± 3.1	89.5 ± 2.9	88.8 ± 3.3	89.3 ± 3.6	88.9 ± 3.2	89.2 ± 3.9	89.1 ± 3.8	88.6 ± 2.8
MCH (pg)	Cobalt	30.3 ± 1.5	30.2 ± 1.5	30.3 ± 1.6	30.2 ± 1.6	30.3 ± 1.5	30.3 ± 1.2	30.3 ± 1.2	30.4 ± 1.3
	Placebo	30.5 ± 1.0	30.4 ± 1.0	30.5 ± 1.1	30.5 ± 1.1	30.5 ± 1.1	30.5 ± 1.2	30.6 ± 1.2	30.3 ± 1.1
MCHC (g/dl)	Cobalt	34.9 ± 0.7	34.5 ± 0.6	34.6 ± 0.7	34.6 ± 0.7	34.5 ± 0.6	34.6 ± 0.6	34.4 ± 0.6	34.2 ± 0.3
	Placebo	34.2 ± 0.4	34.0 ± 0.5	34.4 ± 0.4	34.2 ± 0.55	34.29 ± 0.33	34.2 ± 0.6	34.4 ± 0.5	34.2 ± 0.3
Ret% (%)	Cobalt	1.16 ± 0.42	1.25 ± 0.32	1.19 ± 0.33	1.25 ± 0.41	1.22 ± 0.32	1.14 ± 0.34	1.06 ± 0.37	1.06 ± 0.39
	Placebo	1.07 ± 0.15	1.11 ± 0.21	1.12 ± 0.28	1.10 ± 0.25	1.13 ± 0.27	1.08 ± 0.19	1.02 ± 0.19	0.93 ± 0.19
Ret# (10^6^/μl)	Cobalt	0.058 ± 0.022	0.063 ± 0.018	0.059 ± 0.018	0.063 ± 0.024	0.061 ± 0.018	0.058 ± 0.019	0.052 ± 0.020	0.054 ± 0.023
	Placebo	0.053 ± 0.010	0.054 ± 0.011	0.056 ± 0.016	0.055 ± 0.014	0.055 ± 0.016	0.053 ± 0.013	0.049 ± 0.010	0.046 ± 0.010
OFF-Score	Cobalt	87.4 ± 9.5	86.6 ± 10.1	85.4 ± 7.3	85.4 ± 10.2	84.7 ± 9.2	88.1 ± 11.3	86.5 ± 9.8	89.6 ± 8.4
	Placebo	88.7 ± 6.6	85.7 ± 9.3	88.7 ± 6.1	86.6 ± 6.8	84.3 ± 6.8	87.8 ± 8.5	87.5 ± 9.6	91.1 ± 9.6
IRF (%)	Cobalt	5.5 ± 1.6	6.1 ± 1.9	5.4 ± 1.6	6.1 ± 1.7	5.8 ± 2.7	5.0 ± 1.9	5.3 ± 1.7	4.5 ± 1.4
	Placebo	4.9 ± 1.3	5.0 ± 2.2	5.6 ± 1.5	5.4 ± 2.5	5.3 ± 1.6	3.7 ± 1.5	4.0 ± 1.3	4.6 ± 1.4
RDW-SD	Cobalt	39.4 ± 2.3	40.2 ± 2.2	39.7 ± 2.3	39.8 ± 2.0	40.0 ± 2.0	39.7 ± 1.6	39.8 ± 1.2	39.7 ± 1.1
	Placebo	40.5 ± 1.6	40.7 ± 1.6	39.9 ± 1.6	40.5 ± 1.8	39.8 ± 1.5	40.1 ± 2.4	39.8 ± 2.1	40.3 ± 1.8

Presented are means ± standard deviations (SD). MCV, mean erythrocyte volume; MCH, mean erythrocyte hemoglobin content; MCHC, mean hemoglobin concentration; Ret%, reticulocyte percentage; Ret#, reticulocyte number; Off-score, ([Hb] (g/L)−60) × √ (reticulocyte percentage); IRF, immature reticulocyte fraction; RDW-SD, red cell distribution width. Number of subjects 21 days after cobalt administration: cobalt group: n = 12, control group n = 5. Significance of differences from baseline (post-hoc tests):

*, **, *** p < 0.05, 0.01, 0.001; vs. control group:

# *p < 0.05*.

### Performance

**Figure 3:**
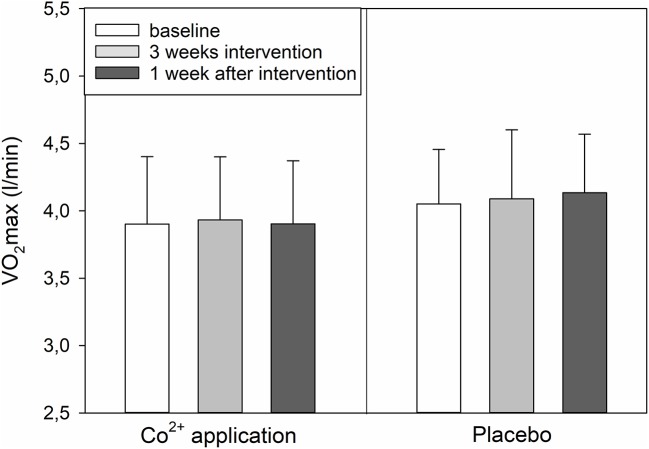
Changes in VO_2max_ immediately and 1 week after the 3-week cobalt application period.

VO_2max_ did not show any changes (Figure 3), while maximum power and time until exhaustion increased over time (*p* < 0.05) without a significant interaction between time and treatment (Table 4). Sub-maximal power at 2 mmol/l and 4 mmol/l blood lactate concentration tended to be affected by CoSupp (interaction between time and treatment: *p* = 0.16, *p* < 0.22; Table 4).

**Table 4:** Performance data after a 3-week low dose Co^2+^ administration.

		**Baseline**	**End of Co^2+^ administration**	**One week after Co^2+^ administration**
VO_2max_ (ml/kg/min)	Cobalt	52.6 ± 6.6	53.1 ± 6.8	52.8 ± 7.4
	Placebo	52.0 ± 4.8	52.6 ± 5.9	53.1 ± 5.0
VO_2max_ (l/min)	Cobalt	3.90 ± 0.50	3.93 ± 0.47	3.90 ± 0.47
	Placebo	4.03 ± 0.43	4.06 ± 0.54	4.15 ± 0.47
Peak power (Watts)	Cobalt	305 ± 36	312 ± 35	315 ± 33
	Placebo	322 ± 28	328 ± 30	328 ± 28
Time until exhaustion (mm:ss)	Cobalt	18:16 ± 2:06	18:37 ± 2:09	18:51 ± 2:00
	Placebo	18:32 ± 1:43	18:51 ± 1:38	18:58 ± 1:44
HR-Peak (bpm)	Cobalt	186 ± 7	187 ± 6	189 ± 6
	Placebo	185 ± 7	184 ± 6	185 ± 5
Lactate-Peak (mmol/l)	Cobalt	11.6 ± 2.4	11.8 ± 2.0	12.0 ± 1.6
	Placebo	11.4 ± 1.7	12.0 ± 1.3	12.1 ± 2.0
Power at 2 mmol/l lactate (Watts)	Cobalt	143 ± 40	148 ± 37	154 ± 4.1
	Placebo	162 ± 25	155 ± 16	154 ± 27
Power at 4 mmol/l lactate (Watts)	Cobalt	212 ± 40	215 ± 36	219 ± 37
	Placebo	222 ± 18	222 ± 22	218 ± 24

*Presented are means ± standard deviations (SD)*.

## Discussion

The most important finding from this study was that CoSupp significantly increased hemoglobin mass, but did not significantly alter aerobic performance. The lack of significant change for the other measured dependent values such as VO_2max_ and [EPO] may likely relate to a greater methodological error in measuring these variables compared to Hbmass. However, the significant change in Hbmass and the tendency for [Hb] and Hct to increase indicates that cobalt supplementation does alter erythropoietic activity.

### Cobalt supplements

Some dietary supplements are promoted as performance-boosting nutrients, although their active ingredients may not be completely declared. Recently, Thevis et al. analyzed 19 commercially available dietary supplements advertised as endurance performance enhancers (Thevis et al., 2016). Eleven of them contained up to 2.6 mg Co^2+^/ml, which was only declared in two of these supplements. The Co^2+^ concentration of the supplement used in this study was declared to be 0.2 mg/ml. However, in almost half of the units we used for supplementation, the real concentration was more than 3 times higher. This data suggests that these kinds of dietary supplements have to be used with caution to avoid any side effects caused by undeclared and/or falsely declared Co^2+^ content. In this study, we did not observe any side effect during and after the 5 mg Co^2+^ administration period. Finley et al. (Finley et al., 2012) determined that a safe life time oral dose (RfD) of cobalt was 0.03 mg Co^2+^ per kg body mass per day (2.1 mg per day for a 70 kg subject). Since during the 3-week cobalt treatment our subjects exceeded this value by 2–2.5-fold without any acute side effects, short-term administration of cobalt in this dose appears to be safe.

### Cobalt excretion in urine

In 2015, cobalt was put on the WADA prohibited list under “S2-Peptide Hormones, Growth Factors, Related Substances, and Mimetics” (WADA, 2017). However, a threshold limit for determining cobalt use by athletes has not been established even though some anti-doping laboratories already test urine samples for their cobalt content.

For the current protocol, the urine Co^2+^ concentrations reported during the 3-week treatment period are in accordance with data from the literature (Krug et al., 2014) and our own experience (Hoffmeister et al., 2018). Because most of the ingested cobalt is not absorbed, and because excretion via feces is the main route for elimination of ingested Co^2+^, urinary excretion accounts for between 10 and 27% of ingested Co^2+^ (Ayala-Fierro et al., 1999; Hoffmeister et al., 2018). The relatively high inter-individual variation of urine Co^2+^ observed in this study may therefore be attributable to differences in gastrointestinal Co^2+^ absorption, while the intra-individual variation in the morning urine samples may also be due to different diuresis.

Because the highest urine Co^2+^ concentrations were found at the end of the administration period, which also exceeded those values measured after a single or 5-day application of the identical Co^2+^ dosage, we may assume that Co^2+^ accumulates in body fluids, which may lead to a stronger erythropoietic response than expected from short-term administration. After cessation of the administration, urine Co^2+^ concentration dropped by approx. 80% within 2 days, which is consistent with the elimination half-life of Co^2+^ after low-dose oral ingestion being between 4 and 12 h (Ayala-Fierro et al., 1999; Tvermoes et al., 2013). Nevertheless, compared to baseline, urine [Co^2+^] was still elevated 2 weeks after cessation, which has implications for establishing threshold limits to combat doping. In a pilot-study, Krug et al. (Krug et al., 2014) calculated 14 ng/ml as a tentative reference limit for urine Co^2+^ concentration, which was determined by multiplying the reference population (*n* = 100 subjects) standard deviation by four. In this study, all participants except one individual exceeded this threshold 1 week after cessation (mean = 43.1 ± 47.1 ng/ml), and some individuals exceeded it even after 2 or 3 weeks. Therefore, using the reference limit mentioned above, it should be possible to detect cheating athletes during doping and for at least 2 weeks after cessation of Co^2+^ ingestion.

### Erythropoietic effect of Co^2+^ administration

Co^2+^ mimics the effects of hypoxic exposure by stabilizing the HIF 1α and 2α subunits and stimulating endogenous erythropoietin synthesis (Jelkmann, 2012). According to Jelkmann it was formerly thought that Co^2+^ could replace the Fe^2+^ in the HIF-α prolyl hydroxylase. Actual data indicate that Co^2+^ may bind to HIF 1α and 2α preventing the interaction with the von-Hippel-Landau tumor suppressor protein (pVHL) (Jelkmann, 2012).

In our recent study (Hoffmeister et al., 2018), we demonstrated that a 5-day Co^2+^ administration of 10 mg/day clearly passes the erythropoietic threshold, as indicated by increased plasma [EPO], a higher percentage of IRF, and decreased plasma ferritin concentration. Smaller dosages as 0.45 mg/day for 2 weeks (Tvermoes et al., 2013) and 1 mg/day for 90 days did not show any effect (Tvermoes et al., 2014) but a Co^2+^ application of 5 mg/day for 5 days showed a tendency toward erythropoietic stimulation (Hoffmeister et al., 2018). The current protocol indicated that the Co^2+^ administration of 5 mg/day for 3 weeks produced clear erythropoietic stimulation, resulting in a 2% increase in Hbmass, which is similar to what has been observed during hypoxic training camps lasting 200 h at an altitude of ~2,000 m (Garvican-Lewis et al., 2016).

To our knowledge, there have been no reports on [EPO] following long-term Co^2+^ application making comparison of our results to those in the literature difficult. In hypoxia, [EPO] increases within 2 days and decreases within a few days to almost baseline values (Wehrlin et al., 2006; Wachsmuth et al., 2013). It is possible that a similar time course occurred in our study but we did not make measurements of [EPO] during the first few days of CoSupp. These measurements were not made since they would require additional blood sampling which might have confounded the interpretation of the erythropoietic stimulus.

The drop in ferritin in both the treatment and the placebo groups was unexpected but might be due to the amount of blood lost as result of the blood sampling required as part of the experimental protocol. A total of 108 ml of blood was required for the analysis of blood parameters which may have altered the iron status of both groups (Wachsmuth et al., 2015). This highlights the need for experimental protocols where Hbmass is the key dependent variable to consider the confounding factor of blood sampling on this parameter.

### Performance data

Although suppliers of nutritional supplements claim enhanced endurance performance from Co^2+^ supplementation, this hypothesis has not been verified in the scientific literature. In a meta-analysis of 145 elite athletes, Saunders et al. (Saunders et al., 2013) found a 3% increase in Hbmass and VO_2max_ after hypoxic exposure (“live high–train high” and “live high–train low” protocols). The correlation between these changes was, however, weak, and it explained less than one-sixth of the variation, indicating that, apart from increased Hbmass, other factors must also be important in causing the elevated VO_2max_ after altitude training (Saunders et al., 2013). In this study, we found a close relationship between baseline Hbmass and VO_2max_ (*r* = 0.775, *y* = 3.3x + 1,121). The slope of the regression line of 3.3 indicates that a change in Hbmass by 1 gram results in a change of VO_2max_ by 3.3 ml/min, which agrees closely with other data in the literature (4 ml/min) (Schmidt and Prommer, 2010).

The change in Hbmass of ~20 g in this study should result in an increase in VO_2max_ of ~60 ml/min, corresponding to 1.5%. Such small differences, however, are very difficult to detect with the commonly used spirometry systems; also, in our laboratory, the methodological noise (typical error) for VO_2max_ determination using incremental cycle ergometer tests is 2.5%. In addition, there is probably a variability in individual response to the cobalt absorption, cobalt clearance, and interference with HIFα as well as the dependency of VO_2max_ on Hbmass. This is supported by the tendency for significant relationship (*r* = 0.40, *p* = 0.1) for changes in Hbmass and changes in VO_2max_. Also the tendencies for increased power at lactate concentrations of 2 mmol/l and 4 mmol/l (Table 4) hint to a performance modulating effect of cobalt.

## Conclusion

We found that 5 mg/day Co^2+^ administration for 3 weeks exceeds the erythropoietic threshold in young leisure-trained men, leading to increased Hbmass and a tendency to improved endurance performance. The urine Co^2+^ concentration greatly exceeded the baseline values during the administration period, and the elevated concentration lasted for at least 1 week after cessation. Concerning the anti-doping fight, it seems to be feasible to establish upper threshold limits for urine cobalt concentrations.

## Author contributions

TH, DS, MT, WB, and WS conceived the study. TH, DS, NW, OK, HG, WB, and WS contributed to data collection, with TH, WB, and WS performing all statistical analyses. TH, WB, and WS drafted the manuscript, to which DS, NW, OK, HG, and MT then contributed. All authors read and approved the final version of the manuscript. TH, DS, NW, OK, HG, MT, WB, and WS agree to be accountable for all aspects of the work in ensuring that questions related to the accuracy or integrity of any part of the work are appropriately investigated and resolved.

### Conflict of interest statement

WS is a managing partner of the company Blood tec GmbH, but he is unaware of any direct or indirect conflict of interest with the contents of this paper. The remaining authors declare that the research was conducted in the absence of any commercial or financial relationships that could be constructed as a potential conflict of interest.
